# Circulating tumour cells predict recurrences and survival in head and neck squamous cell carcinoma patients

**DOI:** 10.1007/s00018-024-05269-1

**Published:** 2024-05-23

**Authors:** Xi Zhang, Chameera Ekanayake Weeramange, Brett G. M. Hughes, Sarju Vasani, Zhen Yu Liu, Majid Warkiani, Gunter Hartel, Rahul Ladwa, Jean Paul Thiery, Liz Kenny, Omar Breik, Chamindie Punyadeera

**Affiliations:** 1 Saliva & Liquid Biopsy Translational Laboratory, Griffith Institute for Drug Discovery, Griffith University, 46, Don Young Rd, Queensland, QLD 4111 Australia; 2 Menzies Health Institute Queensland, School of Medical Science, Griffith University, Gold Coast Campus, Gold coast, QLD Australia; 3 Cancer Care Services, Royal Brisbane and Women’s Hospital, Herston, QLD Australia; 4 School of Medicine, University of Queensland, St Lucia, QLD Australia; 5 Department of Otolaryngology, Royal Brisbane and Women’s Hospital, Herston, QLD Australia; 6 School of Biomedical Engineering, Center for Health Technologies (CHT) & Institute for Biomedical Materials & Devices (IBMD), University of Technology Sydney, Sydney, Australia; 7 QIMR Berghofer Medical Research Institute, Brisbane, QLD Australia; 8 Dept of medical oncology, Princess Alexandra Hospital, Woolloongabba, Australia; 9 Guangzhou Laboratory, Guangzhou International Bio Island, No. 9 XingDaoHuanBei Road, Guangzhou, 510005 Guangdong Province China; 10 Translational Research Institute, Woolloongabba, Brisbane, Australia

**Keywords:** Head and neck squamous cell carcinoma, Circulating tumour cells, Prognostic, Biomarker, Recurrence, Monitoring, Risk assessment

## Abstract

Patients with head and neck squamous cell carcinoma (HNSCC) are at a high risk of developing recurrence and secondary cancers. This study evaluates the prognostic and surveillance utilities of circulating tumour cells (CTCs) in HNSCC. A total of 154 HNSCC patients were recruited and followed up for 4.5 years. Blood samples were collected at baseline and follow-up. CTCs were isolated using a spiral microfluid device. Recurrence and death due to cancer were assessed during the follow-up period. In patients with HNSCC, the presence of CTCs at baseline was a predictor of recurrence (OR = 8.40, *p* < 0.0001) and death (OR= ∞, *p* < 0.0001). Patients with CTCs at baseline had poor survival outcomes (*p* < 0.0001). Additionally, our study found that patients with CTCs in a follow-up appointment were 2.5 times more likely to experience recurrence or death from HNSCC (*p* < 0.05) prior to their next clinical visit. Our study highlights the prognostic and monitoring utilities of CTCs’ in HNSCC patients. Early identification of CTCs facilitates precise risk assessment, guiding treatment choices and ultimately enhancing patient outcomes.

**Supplementary Information:**

The online version contains supplementary material available at 10.1007/s00018-024-05269-1.

## Background

Head and neck squamous cell carcinoma (HNSCC) is one of the most common and deadliest cancers accounting for an estimated 930,000 new cases and 470,000 deaths worldwide in 2020 [1]. The substantial mortality rate (~ 50%) can be mainly attributed to high relapse rates of HNSCC patients either locoregional or distant [2]. Clinical examination, alongside standard imaging-based strategies such as fluorodeoxyglucose-positron emission tomography-computed tomography (FDG PET-CT) is not ideal for use in routine disease monitoring, due to the to the requirement for frequent monitoring (once every 1–3 month) combined with the limited availability of specialized clinical imaging services [3]. As such, there is an unmet clinical need to establish biomarkers that are capable of prognostication and monitoring, to allow early diagnosis of relapse at a stage where salvage is more likely to be successful.

Circulating tumour cells (CTCs) serve as a pivotal marker in addressing this unmet clinical need. CTCs originate from primary and metastatic tumours, following intravasation into either the bloodstream or lymphatic systems and subsequently after extravasation establishing metastases at secondary sites [4]. Mechanism of CTC generation varies, but CTCs can be used as potential biomarkers to improve outcomes in patients with cancer [4]. This minimally invasive liquid biopsy-based cancer detection holds great potential for the management and longitudinal characterisation of rapidly evolving tumour traits [4–9]. In some cancer patients, CTCs can be detected at an early stage of cancer development and subsequently throughout the course of the disease enabling real-time monitoring of disease burden, and early detection of cancer relapse [10]. As the precursor of metastasis, CTCs often correlate with the risk of cancer dissemination and hence are associated with patient outcomes [11, 12]. The prognostic utility of CTCs has been established across many types of cancers such as lung [13], prostate [14], breast [15], glioblastoma [16], and gastrointestinal cancer [17] where studies indicate that high CTC counts are frequently indicative of poor prognosis. Although such associations in HNSCC have not been investigated comprehensively, available evidence emphasizes potential clinical utility [18–20].

Detection and characterization of rare CTCs from cancer patients’ blood rely on their biological and physical properties. Several techniques have been developed to enrich CTCs, each of which has inherent advantages and disadvantages [21–23]. Among the methods developed, the CELLSEARCH® system (Menarini Silicon Biosystems, Bologna, Italy) which is based on the capture of cells expressing the epithelial cell adhesion molecule (EpCAM) is the first United States Food and Drug Administration-approved method [12, 24]. Although EpCAM-based CTC detection has shown great promise across many types of cancers, this method may not capture CTCs which have undergone an epithelial-to-mesenchymal transition (EMT) since they may not express EpCAM [9, 18, 25].

EMT significantly influences cancer cell metastasis, highlighting the importance of identifying and assessing CTCs that have undergone this process for accurate prognosis and disease monitoring [26–28]. Other mechanisms also play a vital role in the generation of CTC. Hyunbin et al. illustrated that cancer cells can undergo detachment and enter the bloodstream through a process known as adherent-to-suspension transition (AST) [29]. We have previously shown that label-free spiral microfluidic technology could reliably be used to detect CTCs from HNSCC patients’ blood with no selection bias for both EMT and AST [30, 31]. The spiral chip is a marker-independent and high-throughput device that utilises hydrodynamic forces present in curvilinear microchannels under low shear-stress conditions for size-based capturing of cells [32]. In a previously published study, we have demonstrated that in HNSCC patients, baseline CTCs predict events at the 13 week post-treatment PET-CT scan data [31]. In the current study, we have employed this innovative CTC isolation technology to systematically investigate the clinical utility of CTCs for HNSCC prognostication and monitoring post-treatment. This study is one of the largest studies to date to evaluate the prognostic and monitoring utility of CTCs in HNSCC.

## Methods

### Study participants

HNSCC patients who were > 18 years of age were included in the study. We excluded patients with salivary gland, thyroid, nasopharynx cancers and metastatic cutaneous malignancies. Patients receiving curative radiotherapy received 70 Gy in 35 fractions and that concurrent systemic therapy was cisplatin either 3 weekly 100 mg/m2 for 3 doses or weekly 40 mg/m2 for 7 doses, or cetuximab 400 mg/m2 loading dose week prior to radiotherapy and 250 mg/m2 weekly for seven weeks concurrently with radiotherapy or surgery were included. All study participants provided informed written consent prior to inclusion in this study. Tumours were staged according to AJCC Cancer Staging Manual 8th Edition [33]. Part of the patients featured in this research has previously been recruited and their samples were utilized for analysis in our earlier publication Part of the study cohort has been published in a in this study was previously recruited and their samples were used for analysis in our previous publication study [31]. The current study involves an extensive monitoring of patients for up to 4.5 years, with the first participant recruited on 23/Feb/2018 and the last clinical information collected on 23/March/2023.

HNSCC patients were grouped into p16 positive oropharyngeal squamous cell carcinoma (OPSCC) and other HNSCCs (oral cavity cancer, p16 negative OPSCC, hypopharyngeal cancer, laryngeal cancer, and cancer with unknown mucosal primary site) based on their clinical and biological characteristics. Following written informed consent, 15 ml blood was collected from HNSCC patients in K2E EDTA (Cat#: 45,505, Greiner Bio-One, Gloucestershire, UK) vacutainers.

### Sample size calculations

A comparison of CTC counts between patients with recurrence or death versus those without has ≥ 90% power with a sample size of 100 patients, assuming that half are responders and that responders have an average CTC count of 1.0 (Poisson rate) and that non-responders have a rate of at least 1.75 using a Poisson-based comparison of rates between the two groups and α level = 0.05.

### Circulating tumour cell isolation and enrichment

The blood samples were transported to the laboratory within a two-hour window of collection and promptly processed upon arrival. To reduce the non-CTC cellular components passing through the spiral microfluidic chip, an initial red blood cell lysis using RBC lysis buffer (cat# 786 − 649, G-Bioscience, MO, USA) was performed as per our previous publications [7, 12, 31]. Subsequently, cells were centrifuged, and cell pellets were resuspended in 10 mL sheath buffer (1xPBS ((ThermoFisher, MA, USA), 2 mM EDTA (Merck, Darmstadt, Germany), 0.5% BSA (Merck)). Next, Tygon® tubing was inserted into the inlet/outlet of the spiral microfluidic chip, and the inlet tubing was connected to a syringe pump. The spiral microfluidic chip was positioned and fixed onto a phase contrast microscope to monitor the fluid flow. The outlet tubing was connected to two sterile 15 ml collection tubes. An initial priming run was performed using the sheath buffer at a flow rate of 1.7 ml/min for 5 min. Patient samples were loaded into 10 ml syringes (Cat# # 51,903, Terumo, Tokyo, Japan) and pumped through the spiral microfluidic chip using the syringe pump at a flow rate of 1.7 mL/min. The outputs were collected and spun down at 500× g for 5 min. The enriched cells were then fixed with 4% PFA for 10 min and spun onto a polylysine-coated glass slide (ThermoFisher).

### Immunofluorescent staining of circulating tumour cells

Immunofluorescence (IF) staining was used to detect CTCs enriched by the spiral microfluidic chip following a previously published method [31]. The performance of the CTC evaluation procedure was previously established. Cytospun samples were briefly washed with PBS and air-dried. Next, the cells were permeabilised with 0.1% Triton-X100 (Merck) for 10 min. After washing the samples with 1xPBS, they were blocked with 10% FBS (ThermoFisher). After washing with PBS, the cells were stained with a cocktail of antibodies containing anti-cytokeratin monoclonal antibody AE1/AE3 conjugated with eFluor™ 570 fluorophore (cat# 41-9003-82, ThermoFisher), anti-CD45 monoclonal antibody conjugated with APC fluorophore (Cat# 340,943, BD Biosciences, NJ, USA), anti-Cell Surface Vimentin (CSV) antibody 84 − 1 conjugated with FITC fluorophore (cat# H00007431-MF08, ThermoFisher, USA) and DAPI. The slides were incubated for 1 h at room temperature, washed 3 times in PBS, coverslipped and imaged under a fluorescent microscope (Zeiss Imager Z2, Zeiss, Oberkochen, Germany).

CTCs were defined as cytokeratin-positive, DAPI-positive cells and CD45-negative cells larger than 10 μm with an intact cell membrane. Cells staining positive for CD45 and DAPI and negative for pan-cytokeratin were determined to be white blood cells and excluded from the analysis. FaDu (cat# HTB-43, purchased from ATCC, Virginia, USA), a HNSCC cell line expressing cytokeratin was used as a positive control for the immunostaining procedures. Scanning of the CTC slides was performed on the Zeiss Axio Z2 microscope and sequential images were captured after fluorescent staining. A multi-exposure protocol was used to detect the signals. A tile scanning mode was set-up to image the whole surface area of the slides. The Zen software was used to interrogate the images and constrained iterative algorithms were used for image deconvolution. CTC slides were evaluated by two independent researchers. The effectiveness of the CTC evaluation procedure was previously established [31]. The CTC count in the blood samples was normalised to 15mL blood sample. CSV expression intensities were calculated by averaging the FITC signal within the region of interest (ROI) and then dividing it by the average signal from the DAPI staining within the same ROI.

### Statistical analysis

All statistical analyses were performed using GraphPad Prism 8 (GraphPad Software Inc., La Jolla, CA, USA), R (R Development Core Team. Vienna, Austria) and JMP Pro (v16.1 SAS Institute, Cary NC, USA). GraphPad was used to analyse baseline clinical characteristics. Continuous variables were assessed for normality using the Shapiro–Wilk test. If an approximate normal distribution could not be achieved, Kruskal–Wallis tests with Dunn’s multiple comparisons tests were performed to compare multiple groups. The number of CTCs present in a blood sample was modelled as Poisson variables using generalised linear models with a Poisson distribution to generate 95% confidence intervals for the mean counts (i.e. Poisson Rates) and provide comparisons between groups. Logistic regression was used to model treatment response versus clinical and biomarker variables. Contingency tables were analysed with likelihood ratio chi-square tests as these produce p-values equivalent to logistic regression.

## Results

### Participant characteristics

In total, 217 patients with locoregionally advanced HNSCC undergoing curative treatment were recruited to this study. Out of these 217 patients, 63 did not provide follow-up blood samples due to several reasons (e.g. COVID-19 related access issues, failure to attend clinical appointments) and, as such, were excluded from the analysis. The clinical characteristics of the remaining participants (*n* = 154) are listed in Table 1. There are a total of 143 patients were enrolled and had their first sample collection at baseline timepoint, and 11 patients were enrolled and had their first samples collected at disease recurrence. The workflow of patient recruitment is documented in supplementary Fig. 1.

**Table 1:** Patients characteristics

Patients	P16 Positive	Other HNSCC	Overall
	97 (63.0%)	57 (37.0%)	154
Gender			
Male	80 (82.5%)	44 (77.2%)	124
Female	17 (17.5%)	13 (22.8%)	30
Age			
< 60	50 (51.5%)	21 (36.8%)	71
≥ 60	47 (48.5%)	36 (63.2%)	83
Primary site			
Oral Cavity	1 (1.0%)	39 (68.4%)	40
Oropharynx	92 (94.8%)	9 (15.8%)	101
Hypopharynx	1 (1.0%)	1 (1.8%)	2
Larynx	1 (1.0%)	6 (10.5%)	7
Unknown	2 (2.1%)	2 (3.5%)	4
Tumour stage (AJCC 8th Ed)			
I	57 (58.8%)	10 (17.5%)	67
II	23 (23.7%)	6 (10.5%)	29
III	15 (15.5%)	11 (19.3%)	26
IVa or IVb	2 (2.1%)	30 (52.6%)	32
Nodal stage (AJCC 8th Ed)			
0	11 (11.3%)	27 (47.7%)	38
1	63 (64.9%)	9 (15.8%)	72
2	22 (22.7%)	15 (26.3%)	37
3	1 (1.0%)	6 (10.5%)	7
Baseline CTC status			
CTC positive	35 (36.1%)	19 (33.3%)	54
CTC negative	62 (63.9%)	38 (66.7%)	100
Treatment			
CRT + concurrent systemic therapy	76 (78.4%)	10 (17.5%)	86
CRT alone	3 (3.1%)	1 (1.8%)	4
Palliative treatment	0 (0.0%)	1 (1.8%)	1
Surgery	12 (12.4%)	33 (57.9%)	45
Surgery + CRT + concurrent systemic therapy	6 (6.2%)	12 (21.1%)	18

Abbreviation: HNSCC- Head and neck squamous cell cancer; CRT – Curative chemoradiotherapy; AJCC – American Joint Committee on Cancer; CTC – Circulating tumour cells

### CTC enumeration in blood samples collected from HNSCC

A total of 347 blood samples from baseline, 3 months, 6 months, 1 year and 2 years post treatment were collected from all patients. We observed no differences in the number of CTCs between p16 positive OPSCC compared with other HNSCCs (one-way ANOVA *p* = 0.1707) across all timepoints. We detected at least 1 CTC in 3 ml blood samples in 157 blood samples out of 347 samples (45.2%). The trend of CTC changes for all patients are summarised in Supplementary Fig. 2.

**Fig. 1:**
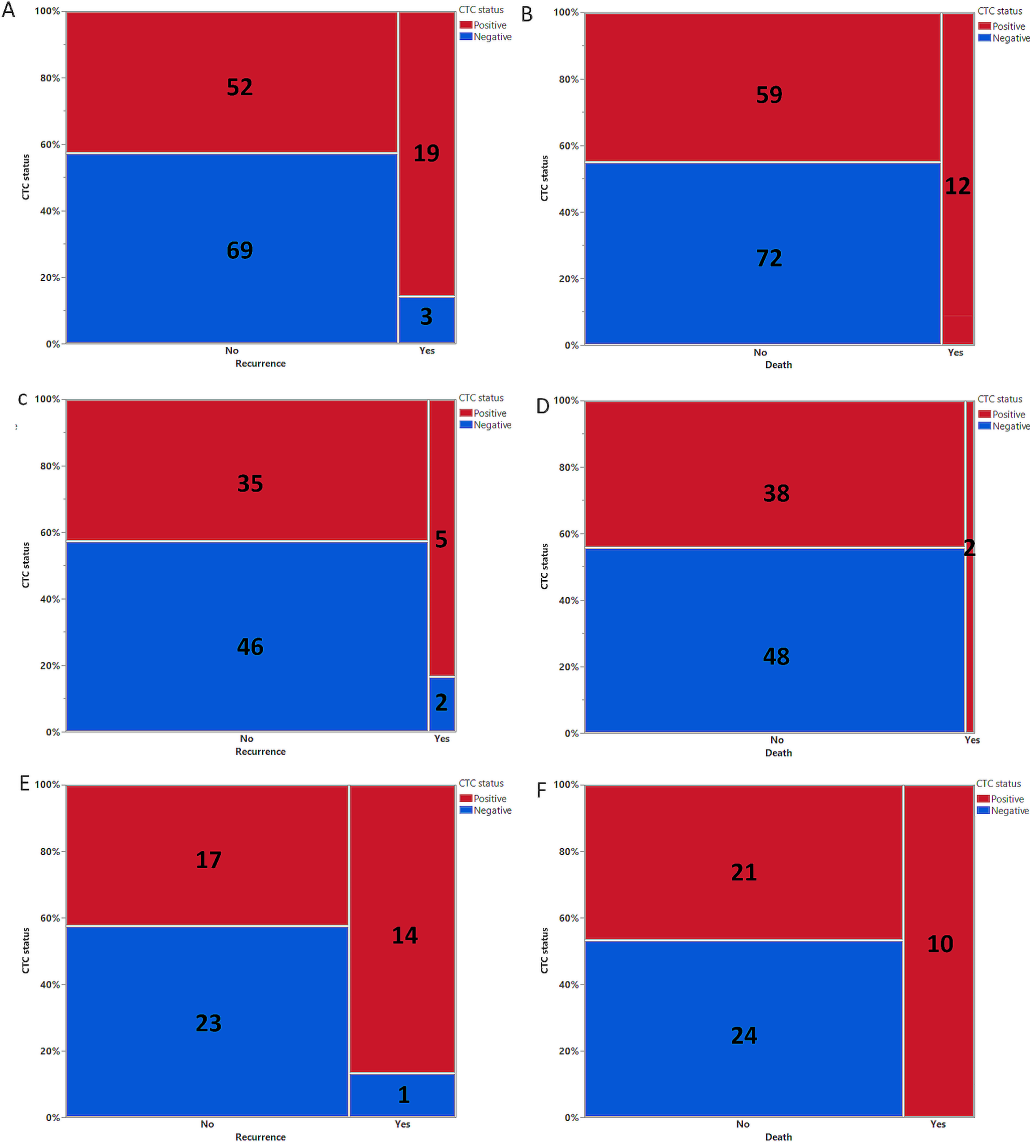
Contingency analysis of circulating tumour cell (CTC) numbers (**A**) in all HNSCC patients with and without recurrence. (**B**) in all head and neck squamous cell carcinoma (HNSCC) patients with and without death. (**C**) in all P16 positive oropharyngeal squamous cell carcinoma (OPSCC) patients with and without recurrence. (**D**) in P16 positive OPSCC patients with and without death. (**E**) in other HNSCC patients with and without recurrence. (**F) in o**ther HNSCC patients with and without death. Number of patients in each category are listed on the corresponding graph

In HNSCC patients who developed recurrences, we found at least 1 CTC at baseline in 19 out of the 22 patients (86.4%), compared to 52 out of 121 patients without recurrence (43.0%) (Odds ratio = 8.40 (95% CI: 2.36–29.92), Chi Square likelihood ratio x^2^ = 15.36, *p* < 0.0001, Fig. 1A). In HNSCC patients who died from cancer, we found all 12 patients (100.0%) had at least 1 CTC in their baseline sample, which was significantly more frequent compared to patients who survived the follow-up period (59 out 131 (45.04%) and had at least 1 CTC (Chi Square likelihood ratio x^2f^ = 17.921, *p* < 0.0001, Fig. 1B).

**Fig. 2:**
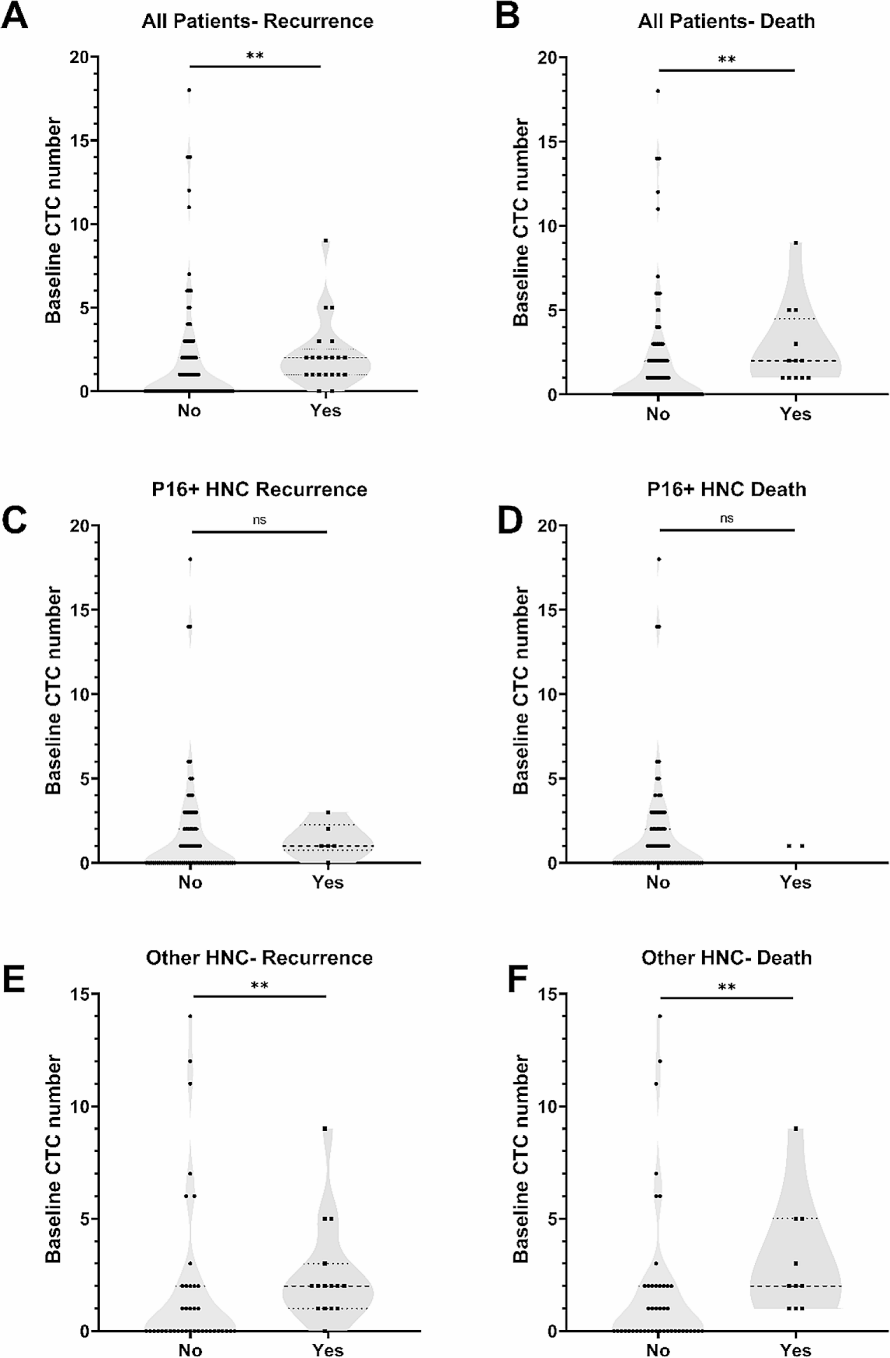
Enumeration of Circulating tumour cell (CTC) numbers in (**A**) in all head and neck squamous cell carcinoma (HNSCC) patients with and without recurrence. (**B**) in all HNSCC patients with and without death. (**C**) in all P16 positive HNSCC patients with and without recurrence. (**D**) in P16 positive oropharyngeal squamous cell carcinoma (OPSCC) patients with and without death. (**E**) in other HNSCC patients with and without recurrence. (**F**) in other HNSCC patients with and without death. P-values are based on comparison of Poisson rates using generalized regression, ns *p* > 0.050, ** *P* < 0.010

Among the patients who did not develop recurrence or death during the follow-up period, the CTC numbers in the baseline blood samples ranged from 0 to 18 CTCs with a mean of 1.64 (Poisson rate, 95% CI 1.06, 2.22), which was significantly lower than in those who had recurrence (CTC numbers ranged from 0 to 9 with a mean of 2.19, 95% CI 1.26–3.12, Fig. 2A, *p* < 0.01). In the 12 patients who died, the baseline CTC number ranged from 1 to 9 with a mean of 2.75 (95% CI 1.19–4.31). This was significantly higher (*p* < 0.01) than the baseline CTC found in patients who did not die, which ranged from 0 to 18, with a mean of 1.63 (95% CI 1.08–2.17, Fig. 2B).

### CTCs predict adverse events in both p16 positive and other HNSCC patients

In patients with P16 positive OPSCC (*n* = 88), there were 7 patients who developed recurrence. We detected at least 1 CTC in baseline blood samples from 5 of these patients (71.4%). No significant difference was observed in the CTC detection rate when compared to P16 positive OPSCC patients without recurrence with only 35 out of 81 patients (43.2%) having at least 1 CTC detected in their baseline sample (Odds ratio = 3.29, 95% CI:0.60-17.94, ChiSquare Likelihood Ratio = 2.098, *p* = 0.14, Fig. 1C) in this group. There were 2 patients with P16-positive OPSCC who died due to cancer, and CTCs were found in both of their baseline samples (100.0%). Out of the 86 patients with P16 positive OPSCC, 38 of them (44.2%) had CTCs in their baseline samples and were still alive. When comparing the P16-positive OPSCC patients who died and those who did not, no significant difference was found (*P* = 0.07, Fig. 1D).

In patients with other HNSCC (*n* = 55), 15 patients (27.3%) had recurrence. Among them, CTCs were detected in baseline samples of 14 patients (93.3%). In the 40 patients who did not have recurrence, CTCs were detected in 17 patients (42.5%). There was a significance difference (*p* < 0.01, Fig. 1E) in CTC detection rate in patients with other HNSCC with and without recurrence. Ten patients with other HNSCC died, and CTCs were detected in all of them (100.0%). In patients with other HNSCC who did not die (*n* = 45), CTCs were detected in 21 patients (46.7%), which was significantly less than in patients who died (*p* < 0.001, Fig. 1F).

### The association between baseline CTC count and patient’s cancer stage and nodal stage

**Fig. 3:**
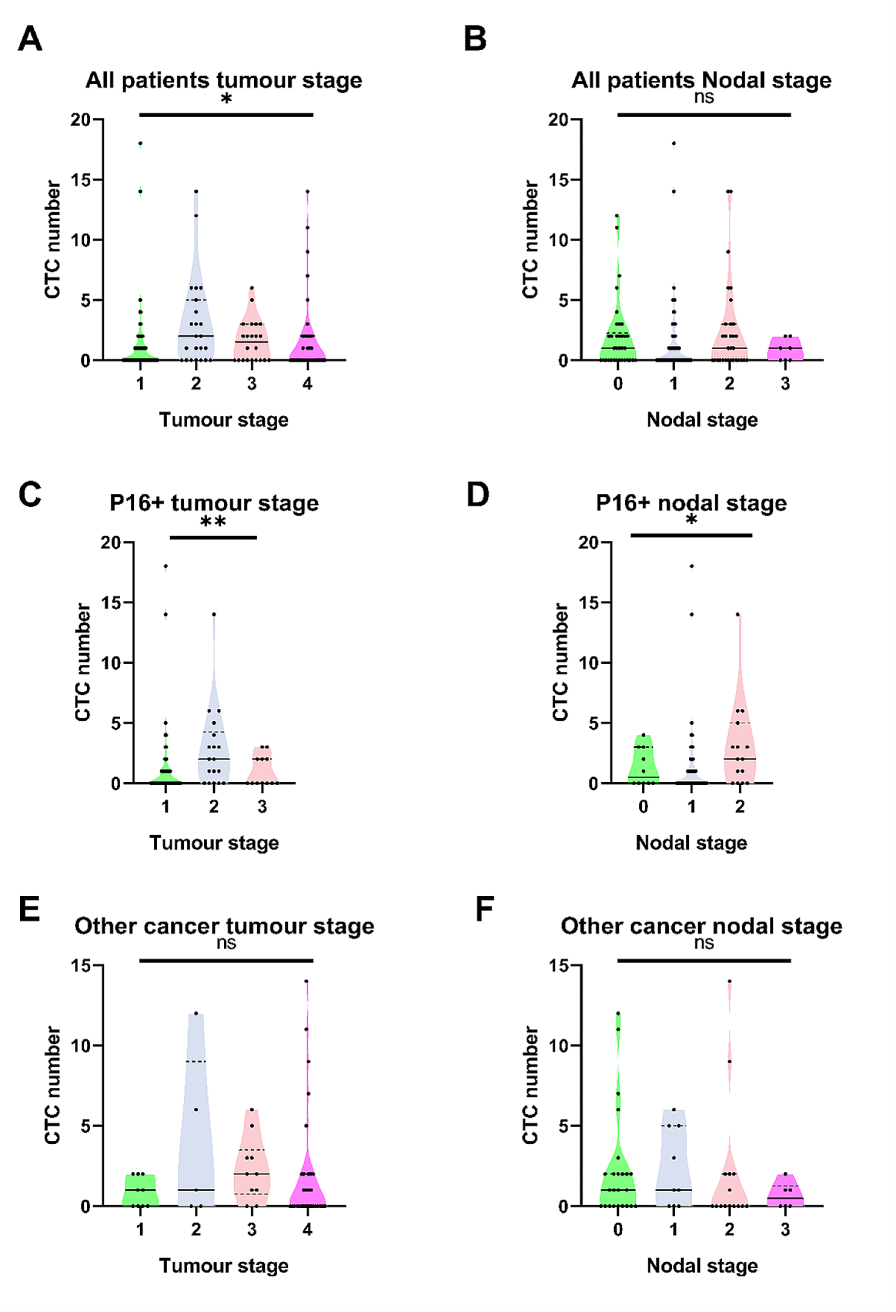
Enumeration of circulating tumour cell (CTC) counts in (**A**) in all head and neck squamous cell carcinoma (HNSCC) patients based on their tumour stage. (**B**) in all HNSCC patients based on their nodal stage. (**C**) in all P16 positive oropharyngeal squamous cell carcinoma (OPSCC) patients based on their tumour stage. (**D**) in P16 positive OPSCC patients based on their nodal stage. (**E**) in other HNSCC patients based on their tumour stage. (**F**) in other HNSCC patients based on their nodal stage. P-values are based on a comparison to Poisson rates using generalized regression, ns *p* > 0.050, * *p* < 0.050, ** *P* < 0.010

We investigated whether there is a correlation between CTC number and the stage of cancer. When the total cohort is considered, there was a significant difference in CTC number when comparing patients with different stage of cancer (*p* < 0.05, Fig. 3A). These significant differences were also observed in the subset of patients with P16 positive OPSCC (Fig. 3B, *p* < 0.01) but not in patients with other types of HNSCC (Fig. 3C, *p* = 0.34). Similarly, we investigated how the nodal staging will affect CTC number. Significant differences were found in patients with P16 positive OPSCC (Fig. 3E, *P* < 0.05), but not in patients with other HNSCC (Fig. 3F, *p* = 0.40).

### Baseline CTC presence predicts time to recurrence and death in patients with HNSCC

**Fig. 4:**
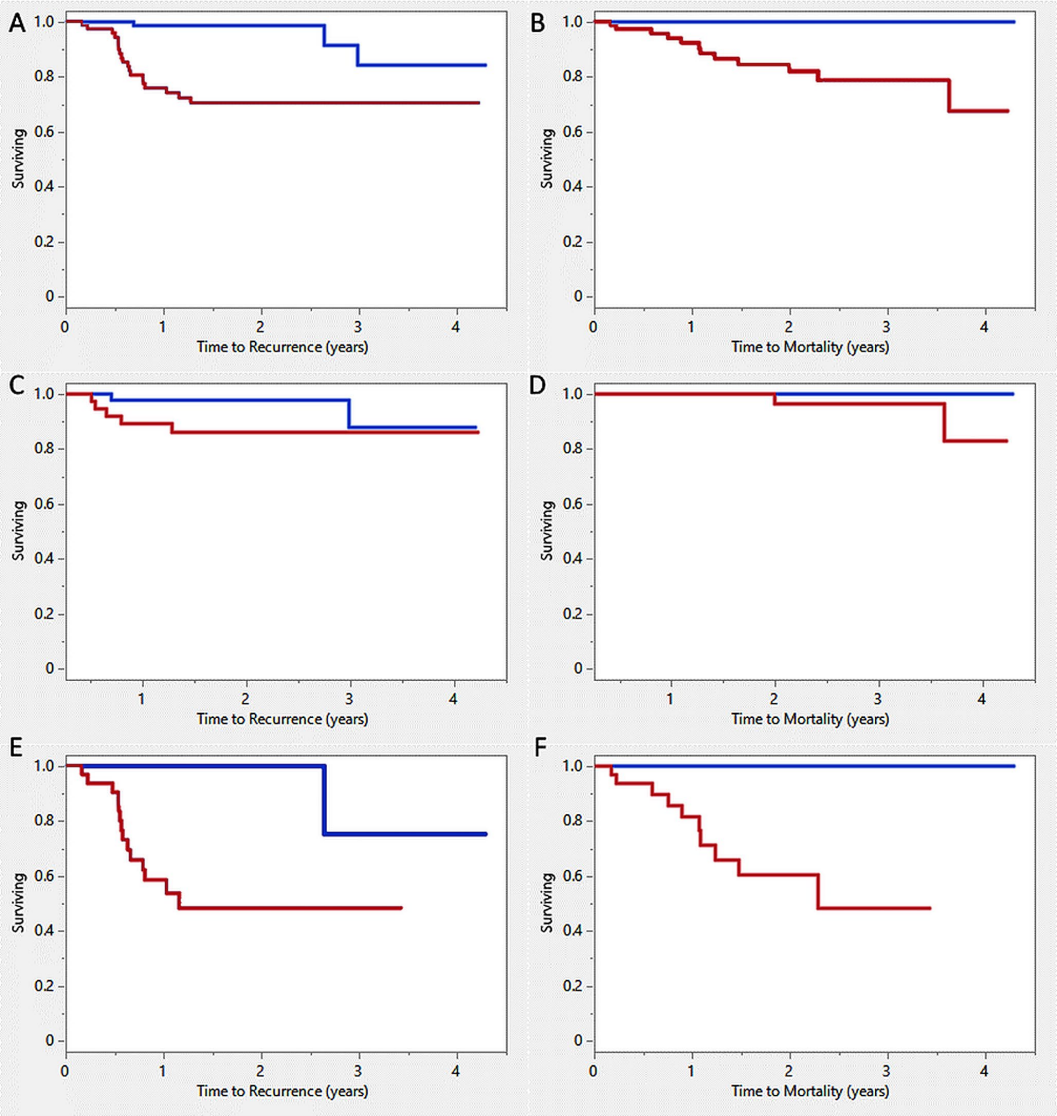
The Kaplan-Meier plot based on baseline circulating tumour cell (CTC) detection (red: CTC+, blue: CTC-) in (**A**) time to recurrence in head and neck squamous cell carcinoma (HNSCC) patients. (**B**) time to mortality due to cancer in HNSCC patients. (**C**) time to recurrence in P16 positive HNSCC patients. (**D**) time to mortality due to cancer in P16 positive oropharyngeal squamous cell carcinoma (OPSCC) patients. (**E**) time to recurrence in other HNSCC patients. (**F**) time to mortality due to cancer in other HNSCC patients

Recurrence occurred in 22 out of 143 HNSCC patients within the follow-up period. Time to the recurrence was significantly shorter (Log-rank test x^2^ = 13.22, *p* < 0.01) in patients with at least 1 baseline CTC (*n* = 22) compared with those with no baseline CTC (*n* = 121, Fig. 4A). Death due to cancer occurred in 12 out of 143 HNSCC patients during the follow-up period. Time to death was significantly shorter (Log-rank test x^2^ = 11.77, *p* < 0.01) in patients with at least 1 CTC in their baseline samples (*n* = 71) compared with those with no CTC found in their baseline samples (*n* = 72, Fig. 4B).

The observed differences in survival and recurrence in HNSCC patients with and without CTCs were mainly driven by patients with other HNSCC (Fig. 4E, F) as no significant survival differences were found among patients with P16 + ve HNSCC (Fig. 4C, D). There were no statistically significant differences in time to recurrence (Log-rank test x^2^ = 1.53, *p* = 0.22, Fig. 4C) and time to death (Log-rank test x^2^ = 1.21, *p* = 0.27, Fig. 4D) in P16 positive HNSCC patients with or without baseline CTCs. In contrast, patients with other type of HNSCC who had baseline CTCs showed a significantly higher cumulative risk of experiencing recurrence (Log-rank test x^2^ = 12.70, *p* = 0 < 0.01, Fig. 4E) and death (Log-rank test x^2^ = 13.60, *p* < 0.01, Fig. 4F).

### Baseline CTCs are independent predictors of recurrence and deaths adjusted for (tumour stages and nodal invasion)

We investigated whether the baseline CTCs are independent predictors of recurrence and death when adjusted with patients’ tumour staging and nodal invasion status (N category). For recurrence, we evaluated their contribution towards classifying patients with and without recurrence using the effect likelihood ratio tests analysis (Table 2). Our results demonstrate that CTC number is an independent factor for determining recurrence even when adjusted for a patients’ cancer stage and/ or N category. Similarly for death, baseline CTC number was shown to be an independent predictor after adjusted for patients’ cancer stage or N category (Table 2).

**Table 2:** Effect likelihood ratio tests cancer stage, nodal stage (N stage), baseline CTC number (log 2 transformed + 1) to predict recurrence and death

Recurrence	Adjusting for tumour stage				Mortality	Adjusting for tumour stage			
	Term	DF	L-R ChiSquare	Prob > ChiSq	Term		DF	L-R ChiSquare	Prob > ChiSq
	Tumour Stage	3	10.07	0.018*		Tumour Stage	3	13.91	0.0030*
	Log2[CTC number baseline + 1]	1	4.23	0.040*		Log2[CTC number baseline + 1]	1	6.2	0.013*
	Adjusting for nodal stage					Adjusting for nodal stage			
	Term	DF	L-R ChiSquare	Prob > ChiSq		Term	DF	L-R ChiSquare	Prob > ChiSq
	Nodal Stage	3	5.45	0.14		Nodal Stage	3	3.73	0.29
	Log2[CTC number baseline + 1]	1	4.01	0.045*		Log2[CTC number baseline + 1]	1	6.34	0.019*

### A higher expression of CSV is observed on circulating tumour cells derived from patients with recurrence

**Fig. 5:**
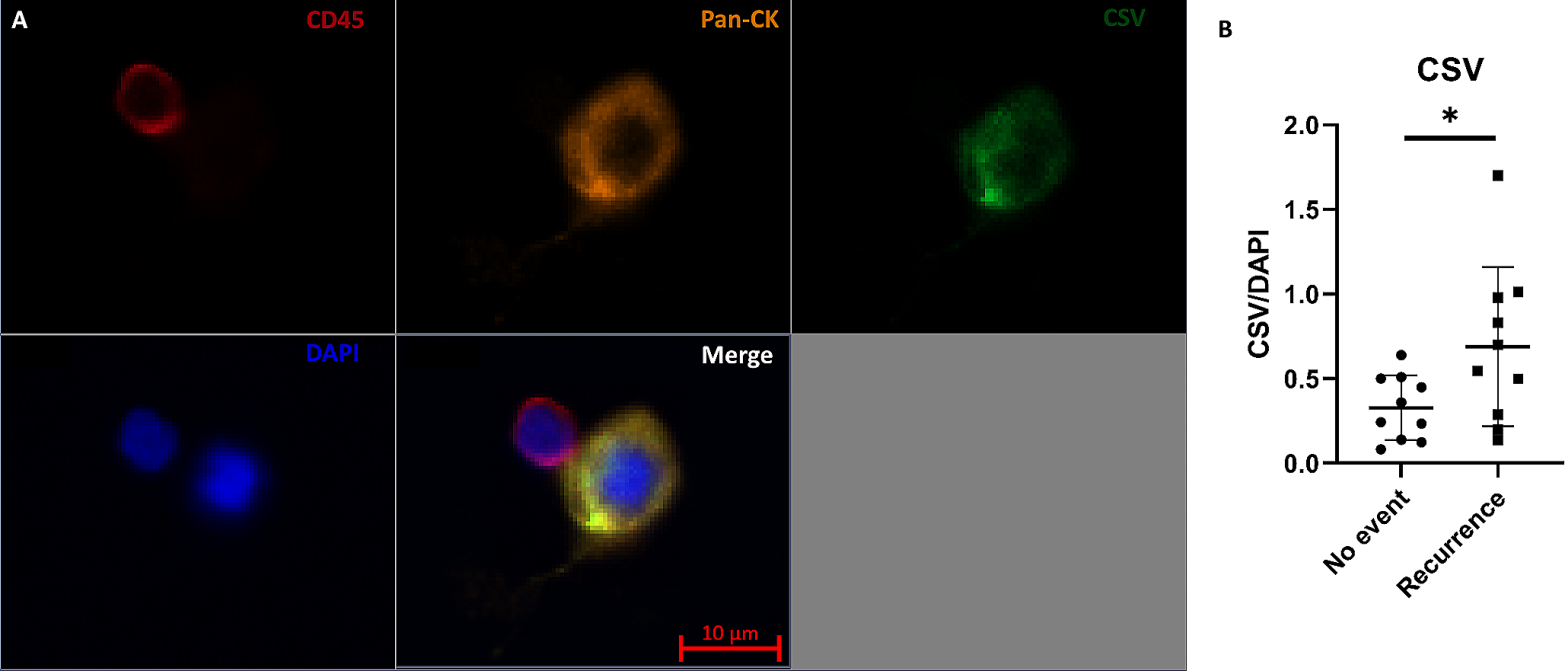
(**A**) An example of circulating tumour cell (CTC) specifically stained with Pan Cytokeratin (orange), Cell surface vimentin (green) and DAPI (blue). White blood cell was stained with CD45 (red) and DAPI. (**B**) Cell surface vimentin expression on CTC isolated from patients with head and neck squamous cell carcinoma (HNSCC) who had had recurrence versus those who had not

We have also evaluated the expression of CSV on CTCs from 20 patients with recurrences and no recurrence (Fig. 5). We observed a significantly higher expression of CSV in HNSCC patients who had recurrence (*n* = 10) compared to those who did not develop recurrences (*n* = 10, *p* < 0.05).

### CTCs in follow-up samples can be used to predict recurrence and death in HNSCC patients

**Fig. 6:**
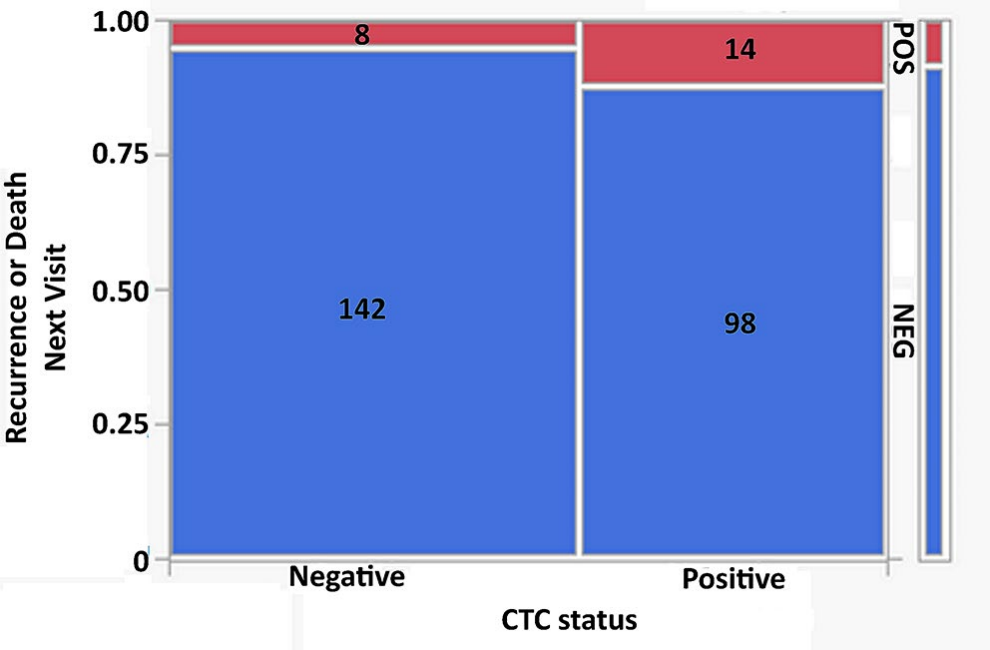
Contingency analysis of circulating tumour cell (CTC) numbers in all head and neck squamous cell carcinoma (HNSCC) patients follow-up time points to predict recurrence or death at next visit. Number of patients in each category are listed on the corresponding graph

We monitored patients for up to 4 years after initial treatment, and found that patients who had CTCs in a follow-up sample were 2.5 times more likely to develop recurrence or die from the cancer in the next clinical visit compared to those who remained CTC free (ChiSquare likelihood ratio = 4.24, *p* < 0.05, Fig. 6), indicating the potential utility of CTCs as a monitoring tool.

### Treatment methods had no effect on either the baseline CTC numbers or follow-up CTC numbers

**Fig. 7:**
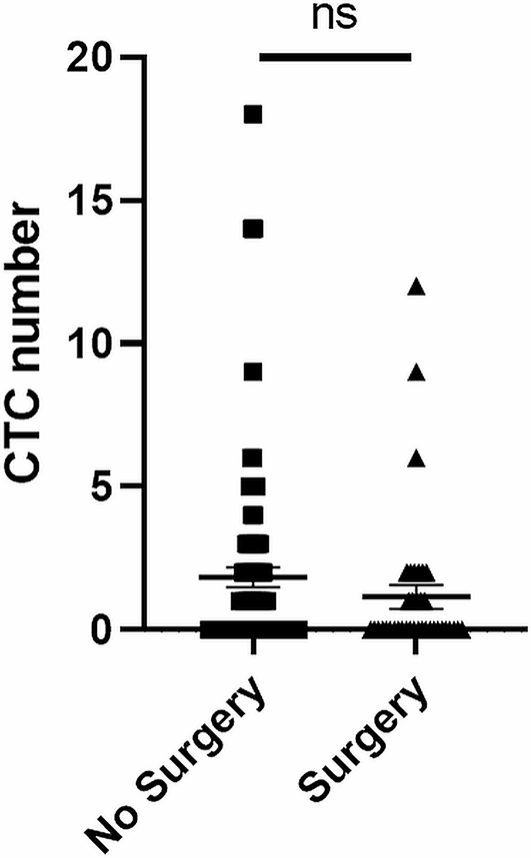
Number of circulating tumour cell (CTC) found in baseline samples collected from head and neck squamous cell carcinoma (HNSCC) patient who undergone surgery treatment and non-surgery treatment

We investigated whether there is an influence of treatment on the detection of CTCs post-treatment. We found no significant differences in CTC numbers both at baseline samples and first follow-up samples between patients who received surgery and those who were non-surgically treated (Fig. 7).

## Discussion

Despite the high relapse rates, currently, there are no established biomarkers for prognostication or monitoring of HNSCC. If adverse events can be predicted in advance, treatment can be customized to improve the outcomes of these patients. CTCs have been demonstrated to be effective markers for disease management in a wide range of cancer types [34]. However, the utility of CTCs for HNSCC management has not been investigated comprehensively. This study reiterates that label-free microfluidic enrichment-based CTC isolation is an efficient method for HNSCC CTC detection as demonstrated by our previous studies [30, 31].

Based on our results, baseline CTC presence was predictive of time to recurrence and death where those who had CTCs at diagnosis experienced recurrence and death within a shorter interval compared to those who lacked CTCs at baseline. When different groups were considered, these associations were only statistically significant for the other HNSCC group. The baseline CTC counts among patients with p16-positive OPSCC was elevated in individuals experiencing recurrence and death compared to those who did not, yet the difference did not reach statistical significance. It is important to highlight the number of recurrences and deaths in the p16 OPSCC group was limited (7 and 2 respectively out of 88) compared to other HNSCC group (15 and 10 respectively out of 55). The predictive value of CTCs in p16 OPSCC, therefore, needs to be investigated in a larger cohort of patients.

Many studies have suggested that the baseline CTC counts can offer valuable insights into cancer prognosis. According to the studies conducted by Magbanua, et al., Pang, et al. and Matikas, et al. CTC presence at baseline sample is associated with shorter overall survival and early recurrence in breast cancer patients [35–37]. Further, several studies have reported that a higher counts of baseline CTCs indicates poor outcomes in prostate cancer patients [38–41]. Similar findings have also been reported for colorectal cancer patients by Silva, et al. [39]. We previously demonstrated that baseline CTC count is associated with treatment outcomes of HNSCC patients determined by 13th week post-treatment PET CT [42]. The current study demonstrates that baseline CTCs are not only predicting treatment response but are also linked with long-term outcomes of HNSCC patients. Confirming that baseline CTCs can provide additional predictive value, the associations observed remained significant even after adjusting for covariates including the TNM stage of the patients. In addition to the associations with baseline CTCs, we also observed that the presence of CTCs in post-treatment follow-up samples indicates a higher risk of experiencing adverse events. According to our findings, HNSCC patients who had post-treatment follow-up CTCs had a 2.5 times higher risk of experiencing recurrence or death by the next clinical visit. This highlights that CTCs can also play an essential role as a monitoring tool for HNSCC. Consistent with our observations, several previous studies have reported th e utility of CTCs for disease surveillance. Gorges, et al. reported that decreasing CTC counts post-treatment is indicative of good treatment response in lung cancer patients [43]. According to Silva, et al., colorectal cancer patients with higher post-treatment CTCs also have poor outcomes [44]. Similarly, Wang, et al. and Lozano, et al. reported that high post-treatment CTC counts are indicative of unfavourable outcomes for prostate cancer patients [40, 41]. Several studies considering breast cancer patients have reported complementary findings [35, 37]. Multiple studies have investigated the relationship between the number of CTC and CTC changes and treatment response in HNSCC, indicating their potential clinical utility as biomarkers for treatment efficacy. Research conducted by Hristozova et al. found a significant reduction in CTC counts four weeks after chemoradiation in HNSCC patients [45]. Similarly, Baa et al. observed a decrease in median CTC counts in patients who responded to treatment, whereas non-responders saw an increase three months post-treatment [46]. Onidani, et al. demonstrates that CTCs can be used to detect dynamic molecular changes following treatment in 10 HNC patients [47]. Zhang et al. discovered in patient with nasopharyngeal carcinoma receiving chemotherapy, not only did the CTC count decreased, but also the frequency of aneuploidy of chromosome 8 in CTCs were also higher post treatment [48]. These findings suggest the broad applicability of CTC in monitoring in response to treatment.

We previously reported that baseline CTCs with mesenchymal characteristics indicate poor treatment response determined by the 13th week post-treatment PET-CT [42]. In the current study, we further investigated these associations by comparing CSV expression in CTCs between recurrent and non-recurrent HNSCC patients. We observed a significant difference in CSV expression between these groups where recurrent patients tend to have EMT-transformed CTCs, determined by high CSV expression, compared to those without events. Tumour cells with hybrid epithelial and mesenchymal phenotypes have been reported to have improved plasticity and have been identified to be linked with poor prognosis in cancer patients [49–52]. Therefore, we believe that secondary EMT scoring using a mesenchymal marker such as CSV may improve the prognostic utility of CTCs. In addition to these associations, we also identified baseline CTC counts of p16-positive OPSCC patients to be associated with the stage of their cancer and lymph node involvement. However, these associations were not observed for the other HNSCC group. Further, we observed an overall reduction in post-treatment CTC counts in patients who underwent surgery. However, no statistically significant association was observed between the treatment approach and post-treatment CTC counts.

In summary, the findings of this study indicate that the presence and count of CTCs at baseline can be used as an independent predictor of HNSCC recurrence and mortality. Furthermore, we observed that patients who have CTCs in follow-up blood samples are at a higher risk of experiencing adverse events compared to those who lack CTCs. Hence, we posit that CTCs can serve as valuable prognostic and monitoring markers for HNSCC.

Limitations.

We have acknowledged in our manuscript that the sample size of P16-positive OPSCC patients who experienced an event during the study was relatively small, which is not surprising and this may potentially diminish the statistical significance of our findings. Consequently, future research with a larger cohort and extended follow-up is essential to ascertain the predictive value of circulating tumour cells (CTCs) for disease progression in this patient group. Additionally, the limited sample size may have constrained our ability to discern significant variances in CTC counts across different disease stages, indicating a need for broader studies with more comprehensive staging data to corroborate these results and deepen our understanding of the correlation between CTC levels and disease progression in HNSCC patients. Despite these limitations, the significant outcomes that we have observed underscore the promise of CTC analysis as an adjunct tool to traditional prognostic methods for HNSCC. However, more multi-centre clinical trials are needed to fully explore CTCs’ role in refining treatment strategies and forecasting treatment responses. Due to the COVID-19 pandemic, several follow-up samples could not be collected.

## Conclusion

This study demonstrates the promising prognostic and monitoring capabilities of CTC in HNSCC, underscoring its potential value. The data demonstrated that the CTC numbers at baseline was correlated with the patients’ cancer tumour staging. Our data also indicates that CTC presence at baseline independently predicts HNSCC recurrence and death, particularly in patients with P16 positive OPSCC. Moreover, patients showing CTCs in subsequent samples faced elevated risks of recurrence or death. This suggests that CTCs can offer earlier intervention and more aggressive treatment strategies in HNSCC, thus improving patient outcomes. Despite challenges like isolating rare CTCs and the need for larger sample sizes in future studies, these findings mark a significant step towards enhancing our understanding of HNSCC and refining our approaches to its management.

### Electronic supplementary material

Below is the link to the electronic supplementary material.

Supplementary Material 1

## Data Availability

The datasets generated during and/or analysed during the current study are available from the corresponding author upon reasonable request. Statements & Declarations.
