# Supplementary figures and images for: Circulating tumour cells predict recurrences and survival in head and neck squamous cell carcinoma patients
Source: Cell Mol Life Sci. 2024 May 23;81(1):233. doi: 10.1007/s00018-024-05269-1 (PMC11116312; doi:10.1007/s00018-024-05269-1)

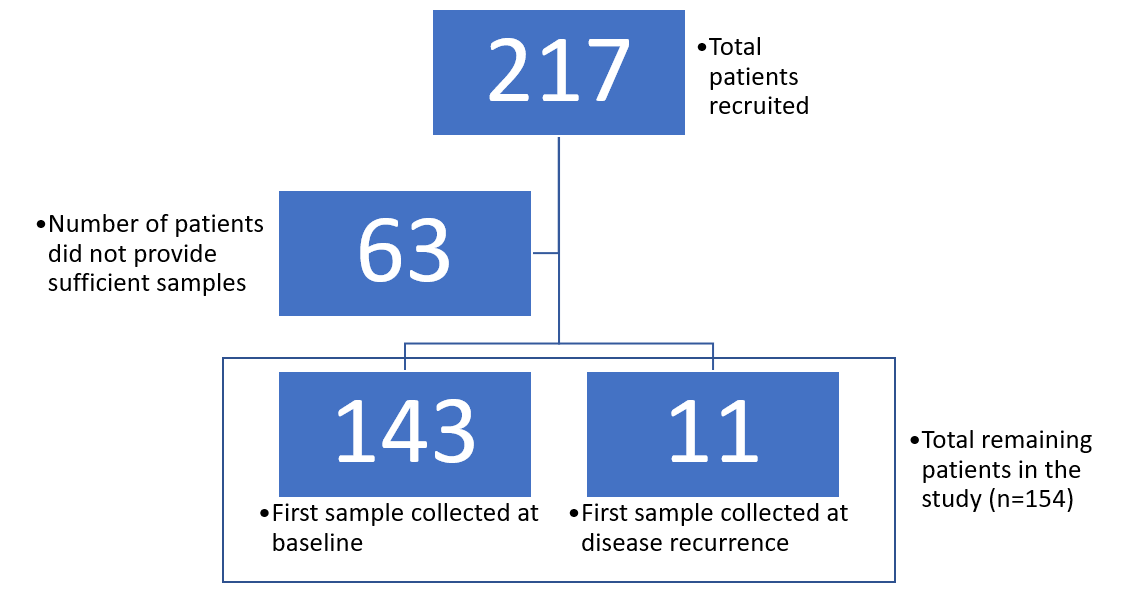

Supplement: Supplementary file 1 — Supplementary Material 1 [file 18_2024_5269_MOESM1_ESM.png]
